# Identifying the Key Elements of Psychologically Safe Workplaces in Healthcare Settings

**DOI:** 10.3390/brainsci13101450

**Published:** 2023-10-11

**Authors:** Karen T. Hallam, Natasha Popovic, Leila Karimi

**Affiliations:** 1 Psychology Department, School of Applied Health, RMIT University, Melbourne, VIC 3000, Australia; karen.hallam2@rmit.edu.au; 2 Institute for Mental and Physical Health and Health Translation, Deakin University, Geelong, VIC 3217, Australia; 3 School of Health Sciences, La Trobe University, Geelong, VIC 3217, Australia; 4 School of Medicine and Healthcare Management, Caucasus University, 0102 Tbilisi, Georgia

**Keywords:** COVID-19, mental health, psychology, frontline, staff, workers, healthcare

## Abstract

Background: Psychological safety is a key concern in the workplace as organisations continue to see increases in psychological injuries that have significant ramifications on individuals and workplaces. The COVID-19 pandemic has exacerbated this issue in healthcare workforces facing extraordinary pressures. This preliminary study aims to enhance our understanding of the factors that healthcare workers value in relation to psychological safety in their respective healthcare settings. Methods: To achieve the research objective, qualitative self-reflection examples were conducted with 12 participants from various health professional backgrounds across public, private, and aged-care settings. The data obtained were thematically analysed using NVivo software (V 12), enabling the identification of key elements associated with psychologically safe workplaces. Results: The results revealed several significant elements that contribute to psychologically safe workplaces in healthcare settings. These elements include effective communication, organisational culture, leadership practices, performance feedback mechanisms, respect among colleagues, staff development opportunities, teamwork, and trust. The findings underscore the critical importance of these foundational elements in fostering psychological safety within healthcare. Conclusion: This study contributes to the existing body of knowledge by specifically identifying the key elements that healthcare workers value in terms of psychological safety. By exploring a wide range of healthcare professionals’ perspectives, this research offers valuable insights into the unique challenges faced by healthcare workforces and the necessary conditions for fostering psychological safety. The implications of these findings are discussed in relation to the lessons they provide for healthcare employers, highlighting the potential for improving workplace wellbeing and performance.

## 1. Introduction

Psychological safety in the workplace is an important element for high-performing teams. Psychological safety typically refers to the creation of workplace cultures that support team members to be themselves as well as to feel that their full selves belong in the company [1]. Notably, psychological safety encourages workers to share ideas, ask questions and place the team’s success as central to their work. In a 2015 study, Ryan (2015) demonstrated that psychologically safe workplaces also encourage mental health and wellbeing [2]. In recent years, the importance of mental health and how it interfaces and impacts workplaces has had increasing attention. The Australian Productivity Commission reports that mental ill health costs Australia AUD 50 billion directly and has a significant AUD 180 billion cost on lost productivity [3]. With the majority of the global population spending one-third of their time at work, workplaces are increasingly being acknowledged as central in terms of both being impacted by distress and playing an important role in maintaining and improving the mental health of their workforces [4]. One of the first steps on this journey is better understanding how to make workplaces psychologically safe.

Psychological safety is an important issue within workplaces as we endeavour to improve the quality of workers’ experiences and avoid mental health tolls on individuals’ personal lives, social experiences, communities and workplaces [5]. The impacts of the COVID-19 pandemic have increased the pressure on Australian workplaces and workers through multiple lockdowns, remote working, and financial hardship for organisations. This has magnified the work pressures and stress faced by both organisations [6,7] and workers [8,9].

The healthcare workforce has been disproportionally impacted by the COVID-19 pandemic and supporting their psychological wellbeing has to be a priority [10]. The research shows that healthcare workers exhibit high rates of pre-existing mental health (MH) disorders [11,12] which can negatively impact on the quality of patient care [13]. Workers within this sector play a vital role in our response to the pandemic and have been faced with significantly increased patient loads, increased personal and family health risks, and prohibitive workplace safety measures including the use of restrictive but essential personal protective equipment. The impacts of the COVID-19 pandemic on the mental health and wellbeing of the healthcare workforce have been extensively highlighted [14,15,16,17,18]. A recent review on COVID-19 implications on neurology residents revealed the impact of the COVID-19 pandemic on experiencing psychological issues [19].

An important study by Smallwood and colleagues during the Australian COVID-19 pandemic highlighted that workplaces and employers had the capacity to improve workers’ coping [20,21]. Specifically, their results indicated that workers who felt supported by their workplaces had reduction in their psychological distress of over 50% including measures of depression, anxiety, burnout, and post-traumatic stress (PTSD). These data highlight that even during the most difficult of times for workforces, strategies, attitudes and approaches by workplaces can significantly impact the mental health and wellbeing of workers. Creating these psychologically safe sectors and workforces is therefore imperative, particularly for the healthcare sector. 

Government initiatives to develop and foster psychologically safe workforces have highlighted the importance of this approach. This includes recent publication of a model code of practice for Australian workforces by SafeWork Australia [21,22]. An important systematic review around the factors that contribute to psychological safety in healthcare workforces was released in 2020 [23].

While it is notable that this review was pre-pandemic, it provides important insights into factors that workplaces might focus on in encouraging psychological safety for their healthcare workforces. These include individual, team and organisational factors. In terms of the organisational factors, the systematic review highlighted that a culture of safety, a focus on continuous improvement, organisational support and familiarity across teams were indicative of workforces that felt more psychologically safe in the workspace. 

Team leaders and managers are at the interface between organisational policies, attitudes and approaches and the workforce. They play an integral role in workforce experiences. Strong leadership and management have been particularly linked to the positive outcomes of psychological safety such as creativity [23,24] and inclusive leadership has been shown to reduce distress in health workers during difficult periods such as the COVID-19 crisis [25]. This highlights the imperative role that leaders and management play in embodying and communicating psychologically safe work management and leadership approaches used with staff that improve the sense of psychological safety for health workers, particularly in the context of the difficult and complex environments that healthcare workforces have faced since COVID-19. 

The aim of this study was to identify the key elements of psychologically safe healthcare workplaces during crises like the COVID-19 pandemic. Positive organisational scholarship (POSH) suggests that focusing on the good, the excellent, and the brilliant is as important as conventional approaches that focus on the negative, the problems, and the failures. Insights gained can then be integrated into health workforce policies and procedures and provide insight to organisations and leaders on how they can better support their workforce during these difficult times.

## 2. Materials and Methods

### 2.1. Participants

This sample included 12 individuals engaged in work in the Australian health workforce during the COVID-19 pandemic. The workers’ roles included clinical physiotherapy, nurse unit management, clinical nurse education, department head of a COVID-19 hotel, aged-care facility management, and administration on healthcare rehabilitation ward. Of the study group, 83.3% were female and 16.7% male with varying age ranges, 16.7% aged 20–30 years, 50% aged 31–40 years and 33.3% aged 41–50 years. Participants were recruited via an opportunistic sample of postgraduate students undertaking a Master of Health Administration qualification at the participating university. The project was fully approved by the participating University Human Research Ethics Committee (Ethics number HEC20381). Students not engaged in a direct healthcare setting or role were excluded form participation in this study. Table 1 below presents the participants’ demographic characteristics.

### 2.2. Design

The research design and methodology within this project included a qualitative cross-sectional research design. The data were analysed using Nvivo (v 12). 

### 2.3. Methodology

Participants were invited via email to participate in this research. The target sample included 50 postgraduate students who were completing an online subject as part of a Master of Health Administration degree. The final participant pool represented the volunteers from this larger cohort for the research. Volunteer participants who met the inclusion criteria of having a current occupational role in the healthcare system were asked to read a Participant Information Form and complete a consent form for the study before their contribution was included in the study.

Participants were asked to report on self-reflective examples of what they perceived as psychologically safe work practices by their management in their healthcare setting. They shared their examples of a psychologically safe practice in workplace via an online subject platform. 

### 2.4. Data Analysis

The qualitative data were collated, and de-identified by the lead researcher. Then, the data were transcribed into word processing software for analysis in NVivo by the student researcher in the health field. Thematic analysis was used to identify the most common themes. Through the qualitative analysis procedure, a number of common themes were extracted with the NVivo software (v 12). These reflected the experiences that the twelve participants had experienced in their healthcare setting.

### 2.5. Ethics Consideration

The project was fully approved by the participating University Human Research Ethics Committee (Ethics number HEC20381). Informed consent was obtained from all participants who volunteered in the study.

## 3. Results

The qualitative data obtained from the 12 participants were organised into the eight most endorsed themes entailing communication, culture, leadership, performance feedback, respect, staff development, teamwork and trust. Together, these themes exhibit how the participants identified what elements are formed in a psychologically safe team.

### 3.1. Communication

The majority of the participants reported that open communication is the main element in a psychologically safe team. This would include management communicating effectively with each worker individually and together as an entire team. This is especially true when teamwork is paramount around planning and development. During the COVID-19 crisis, this capacity was particularly important as processes and systems were constantly changing and evolving in response to new information. One participant states “my team leader had always encouraged open communication in the team meetings. She had regular team meetings with the clinical team and management”. This shows the importance of open communication with staff as a leader, as benefits include feelings such as “all staff felt heard and valued” when ”meetings were effectively managed”. Participants reflected that group discussions allowed for staff to freely share ideas and speak up with the assistance of the team leader. Having clear expectations of these meetings was also important, including the use of an agenda as highlighted by participant 1 in saying “expectations were clear days before the meeting”. Communication continued to be reinforced as important in managers’ use of updates, which were provided, and that “the team leader had always made sure the team members contributed to these meetings and would guide the team where necessary”. Therefore, this atmosphere allows staff to “feel comfortable”, which, in turn, “helped the team to develop strategies and plans” to improve delivery of patient centred care. This participant further mentioned the utilisation of “reflection sessions” which provide an environment to discuss issues, whether good or bad, in a “safe, supportive, non-judgemental and confidential setting”. No topics were ‘off limits’ and it was indicated that management understood staff needs to speak up, feel comfortable and be honest.

### 3.2. Culture

Another theme that arose from psychologically safe teams was team culture, particularly true in healthcare settings when the work is difficult, dangerous and tiring during the COVID-19 crisis. One participant spoke of experienced and educated managers whose team “featured a diverse group of employees with differing levels of experience, skill sets, and cultures. Sharing an office enabled personal relationships to flourish, building interpersonal trust and mutual respect between all staff”. This ensured that all staff were included in the decision making, sharing of ideas and participating in meetings and group discussions. This elicited a sense of safety in the workplace and feeling included and heard by management. Another participant noted that they “built a multi-cultural team”, which, in turn, provided the team with the skill of speaking many distinctive languages.

### 3.3. Leadership

Psychologically safe teams were also characterised by strong leadership, a key element in managing teams in complex and constantly changing environments. One participant stated that “effective leadership in the context of psychological safety requires the mastery of many skills”, a statement particularly true during an international healthcare crisis. A psychologically safe team is shaped by the type of leadership style and qualities such as open communication, encouraging and accommodating preferences.

### 3.4. Performance Feedback

Obtaining feedback on workplace performance is important for staff. Leaders’ capacity and approach to providing feedback is a characteristic of a psychologically safe workplace, particularly in healthcare settings where appropriate and timely feedback are vital to clinical care and workplace safety. For these participants, this type of feedback and collaborative behaviour facilitated staff feeling heard, listened to, acknowledged and feeling supported by management, particularly vital during COVID-19. It was notable that participants reflected that feedback can be delivered in a comfortable manner and increase the staff’s honesty and openness, which, in turn, increases work satisfaction and productivity. One participant also spoke of providing rewards for their outstanding work and those who have not been working adequately and efficiently are on “performance improvement plans and regular counselling and education organised to assist them”. Expectations are understood and, regardless of whether staff are performing well or not, they are supported. 

### 3.5. Respect

Psychologically safe teams were also reflected in the demonstration of values and behaviours showing respect. This includes fostering feelings of acceptance and support for each worker’s qualities, achievements, and abilities. Participant 11 noted that “ In one of the management courses, I learned how important it is to sharpen the tools. I always remembered this and believed in this. I also should mention that kindness and respect also helped to achieve a psychologically safe environment for staff”. It is clearly evident that respecting one another’s opinions, thoughts and feelings, though simple, is a significant factor in team development and fostering psychologically safe work environments. 

It is notable that individuals reported instances of both positive and negative experiences of this respect in their work histories. Individuals noted that these experiences had a significant impact on how they, in turn, behaved. It was noted that when leaders show consistent respect to all workers, workers will then return the respect, improving workplace experiences. A respectful attitude and style also fosters greater comfort with the work environment as noted by one participant, “I felt comfortable during the discussions because they respect me as a valuable team member without regarding my age”. The participant further states that “most of the team members encourage me to share my ideas based on my knowledge. In some cases, where I could not provide my ideas, they allowed me to note and ask my director after the meeting and inform them later” and “there was no interpersonal risk-taking because everyone in the team has the right to participate in this project without care of age or position”. Therefore, the presence of respect continues to be considered a key factor in psychologically safe teams, including those in the healthcare context.

### 3.6. Staff Development

While during crisis periods there may be a focus on merely coping with demand and pressures, participants in this study highlighted that continuous staff development contributed to psychological safety of their teams. Staff development refers to an improvement of skills and knowledge of employees provided through training from the organisation for continuing education, allowing employees to plan their professional growth. It was notable that, during the COVID-19 crisis, these opportunities were fewer; therefore, managers focused on staff personal development and support to increase the sense of a psychological safe environment. 

Other aspects of staff development were noted on the professional aspects of work. A participant noted that “There was an opportunity to implement an improvement board (visual management tool) and a daily huddle for staff to shift quality improvement into our daily practice. The improvement board provided a system that supported learning and continuous improvement at the local level”. This leader ensured that all clinicians, leaders and workers from different areas were included and well aware of this innovation. This ensured that all staff were included and able to share their ideas and opinions. The participant additionally discloses that, during the COVID-19 pandemic, “all leaders, including consultants on ward service were expected to commit time and take turns facilitating sessions to ensure creative ideas and concerns were prioritized”. Leaders themselves were supported to develop as they received “training on how to effectively facilitate a session so that members within the team were valued, heard, the huddles productive, hierarchy was not a barrier, sharing of creative ideas, problem solving and staff taking on ownership of the ideas to allow for working on improvement opportunities”.

### 3.7. Teamwork

An important theme that arose in these reflective examples in light of the COVID-19 pandemic was that of teamwork. Whilst teamwork is an important factor in any context, it was markedly vital during the COVID-19 pandemic in healthcare settings. Successful teamwork is characterised by the development of an environment where ideas are exchanged, and individuals are heard and listened to. Participant 1 noted that during the pandemic “it was a whole team effort and everyone got acknowledged for the work they did. It was a very challenging time both professionally and personally and not just me, but the whole team felt very supported during this time”. One participant adds that during this pandemic, “Staff look after each other and supported to function efficiently and effectively. As a result of good teamwork, complaints have gone down remarkably, and all external complaints are closed, Key Performance Indicators achieved targets, including reducing sick leave and staff are happy to come to work”. These comments highlight that teamwork not only improves team communication, but also improves the capacity to achieve targets, reduces complaints and markedly reduces personal/sick leave, key characteristics of the positive outcomes of psychological safety in the workplace.

The importance of teamwork for psychological safety was enumerated by one participant who reflected that “in my opinion, psychological safety is one of the effective strategies to use to help teamwork or in meetings. To achieve the goal, if you are a manager or leader in one organisation, you should provide psychological safety in the organisation to your team to allow them to feel comfortable or safe to work or share ideas. As a result, they are not only satisfied with their works but also help your organisation grow and succeed”. This supports the importance of teamwork and how it impacts staff wellbeing and engagement. Having a strong connection with other workers enables the opportunity to have trust, be comfortable and respect each other, which leads to greater communication and successful decision making.

### 3.8. Trust

The final theme that encapsulated psychologically safe teams was trust. Many participants reflected how trust is a requirement in order to produce a psychologically safe team; however, not all are able to successfully build it with their staff. Participant 5 had noted this limitation within their own team during the COVID-19 pandemic and had to adopt “a very deliberate approach, focused on building trust specifically with the person who had been requiring the management. On a daily basis I made deliberate efforts to engage on a personal level with this particular staff member who had somewhat been alienated from the rest of the team. I carefully and deliberately communicated in language that was supportive and encouraging and which communicated her value to the team. It slowly became evident that her trust in me and fellow staff was increasing, as was the working relationships improving. She began to be less reactive to fellow team members and would seek me out for assistance and mentoring”. This highlights the role of trust in developing a rapport and re-engaging staff with the wider team. This is particularly imperative in experiences like the above, where staff have had negative experiences in psychologically unsafe workplaces and teams. Participant 8 noted the importance of trust between team members as vital, as well as that between manager and team members during times of crisis like the pandemic. They reported that “that team members are encouraged to be comfortable to involve their team in their work and trust each other. Psychologically safe strategies provides people with an opportunity to share their ideas with their team members with trust and respect to achieve their team goals. Through my experience, I also agree that the trust of team members could allow all members of the team to engage with their works”. Overall, the staff need to feel comfortable and be in an environment where the sharing of ideas is acceptable to all individuals, as well as being heard and listened to, in order to build trust.

## 4. Discussion

The aim of this study was to qualitatively examine the key elements of psychologically safe healthcare workplaces during crises like the COVID-19 pandemic in healthcare settings. Directed by the Positive Organizational Scholarship (POSH) approach, an analysis of the data reveals that during the difficult time over the COVID-19 pandemic when health workforces were under unprecedented pressure, the characteristics that predispose towards a psychologically safe workplace remained relatively constant. These characteristics include open communication, a good workplace and team culture, strong and supportive leadership, timely and thoughtful performance feedback, interpersonal respect, a focus on staff development that reflects the limitations of formal skill development during COVID-19, and the essential characteristics of positive teamwork and shared trust between managers and workers and within teams. 

These results reinforce and support previous findings around psychological safety. For instance, Ryan’s (2015) study stated that current workplaces focus more on the individual experience within the organisational environment instead of focusing on how the organisational environments impact an individual’s health. When employees do not feel comfortable sharing and speaking about initiatives that are not effective, the organisation is not prepared to avoid failure [2]. From this, employees will not be fully committed, resulting in the organisation failing to receive the opportunity to influence the strengths of the employees’ talents. Therefore, it is crucial that employees first feel a sense of belonging, trust and respect, as Maslow’s hierarchy of needs first states that all humans need to have their basic needs met before they are able to reach their full potential. Basic needs entail acceptance. Furthermore, a recent study revealed that teamwork, empowerment and leadership were identified as being crucial elements for a high-performing work system [26]. The thematic analysis revealed how the crucial needs of a worker are required in a workplace in order to reach its full potential, both within the worker’s abilities and achievements and within the organisation. In Ryan’s (2015) study, it was concluded that psychologically safe workplaces encourage the prevention of mental health viability by having a positive workplace wellbeing and mental health [2].

A study conducted in Taiwan involving 320 employees and 80 team leaders on R&D teams, found that greater individual functioning and improved team performance were associated with the implementation of specific leadership practices [27]. The study also found that psychological safety provides lower turnover intention, increased knowledge sharing behaviour and greater task performance. Employees also feel more likely to contribute to developing new services and products, share their ideas and knowledge, find solutions, and are willing to exchange information as they do not fear being seen as weak or disrupting [28]. It is clearly evident that the elements identified in this article establish the basic needs for an individual to provide their full potential in their workplace and this research presents a new way of looking at these requirements and adds to the recent research into psychological safe workplaces in healthcare settings.

Blanchard’s (2020) study reveals that many workforces had to transition to a new work from home system, even though the majority of organisations had never previously supported virtual work [29]. Factors that influenced workers’ productivity and performance include external communications, trust, supervisory support, goal clarity, information sharing and social cohesion. It is clearly evident that these are the necessary needs of an employer, and an employee must have regular check-ins, implement engagement activities, and have informal virtual gatherings in order to strengthen employee engagement in the workplace. Furthermore, other findings revealed that teams performed more effectively, as well as improvements relating to trust and communication [30]. Altogether, these have been found to be the key elements in providing a psychologically safe virtual workplace where workers can prosper. Kahn’s (1990) definition is parallel to this, stating that individuals participated in higher levels of knowledge sharing, which helped the overall productivity of the organisation [31]. 

Kahn’s (1990) findings showed that there are four main factors that influence psychological safety at a workplace, which entail management style, organisational norms, groups dynamics and interpersonal relationships. Having resilient, supportive and clear management has shown an increase in psychological safety, as individuals felt safer due to a sense of control that existed over the work and the absence of fear. The main foundation of leadership is being supported and trust. Leaders who are also encouraging, clear about their expectations and open to trying new things provide employees with a sense of autonomy, ensuring they feel comfortable in making their own decisions [31]. Komori-Glatz’s (2018) study examined the roles of trust and disagreement in multicultural teamwork and the findings show that there is a symbiotic relationship between trust disagreement [32]. This means that the existing high levels of trust and the growing construction of a psychologically safe space allowed team members to disagree as well as challenge one another without any damage to their relationships. This ensured that better decisions were made and that workers can contribute towards success, which also strengthens the team’s individuality.

Valadares (2004) states that healthcare organisations need to advance their resources to ensure there is organisational success. In order for organisations to become empowered and likely to succeed, they must encourage a culture of psychological safety to ensure there is a genuine commitment in their strategies and missions [22]. The implementation of employee initiatives is also crucial; however, these should be derived in a corporate culture and allow employees to feel a sense of safety in order for them to take risks for the improvement of the goals and mission of the organisation [22].

### Limitations and Future Directions

This study was a qualitative investigation conducted during the COVID-19 pandemic with healthcare workers engaged in postgraduate studies in public health. A strength of the study was that it was conducted during the height of the COVID-19 pandemic in Melbourne, Australia, where there were many months of lockdowns and restrictions to curb the impact of the virus. This timeframe meant the research project had to be reflexive and fast to capture this moment. However, this design and implementation also exposed several limitations. For example, this cohort has a particular interest and focus on healthcare systems and experiences and may not represent the experience of healthcare workers who are not engaged in this type of development. Additionally, the cohort for this study was relatively heterogenous in terms of seniority, discipline and healthcare roles. We consider this both a limitation and a strength of the research as it represents a naturalistic cohort that collates information across experiences throughout the healthcare system. Finally, the sample size of 12 was small, and the findings provide insights but not evidence into general trends as the group is small and quantitative results were not collected. 

As with all qualitative research, further investigation and development of qualitative and quantitative research projects are recommended. The results provide intriguing data on the experiences of this cohort during lockdown, a unique moment in our history. While this moment has passed for prospective data collection, there are unique opportunities now to observe whether these same characteristics that remained stable and had yet to be modified during the pandemic to meet the needs of healthcare works and settings continue in post-COVID-19 contexts.

It is worth noting that the characteristics of psychologically safe workplaces identified by healthcare workers during the COVID-19 pandemic are similar to those clearly established in the psychological safety literature. This highlights the stability of the concept and the universality of positive workplace characteristics that improve this goal. With our current evidence indicating the significant impacts of COVID-19 on healthcare workforces in terms of burnout and psychological distress, these results further highlight the importance of utilising these strategies and approaches for organisations and managers. 

## 5. Conclusions

This preliminary study contributes to the existing body of knowledge by specifically identifying the key elements that healthcare workers value in terms of psychological safety. By exploring a wide range of healthcare professionals’ perspectives, this research offers valuable insights into the unique challenges faced by healthcare workforces and the necessary conditions for fostering psychological safety. Encouraging psychological safety in healthcare settings is not easy, particularly during times of enormous healthcare crises and societal upheaval. Despite this, these skills are essential to strengthen and energise health professionals. This not only leads to continuous service improvement, improved clinical outcomes and increased capacity to face significant healthcare burdens, but is also related to improving the experience of these workers. Fundamentally, healthcare workers are the greatest investment and asset of any healthcare workforce and indeed some of the greatest assets within communities, particularly during crises. Interventions and approaches that reduce burnout and focus on improving mental health and wellbeing are important initiatives.

## Figures and Tables

**Table 1:** The participants’ demographic characteristics (*n* = 12).

Gender	Total
Male	2 (16.7%)
Female	10 (83.3%)
Age	
20–30 years	2 (16.7%)
31–40 years	6 (50%)
41–50 years	4 (33.3%)
Place of work	
Private hospital	3 (25%)
Public hospital	7 (58.3%)
Community nursing/aged-care	1 (8.3%)
Other	1 (8.3%)
Profession/work background	
Allied Health	3 (25%)
Management/Leadership	4 (33.3%)
Clinical Educator	1 (8.3%)
Junior Medical staff	1 (8.3%)
Clerical or Administrative	1 (8.3%)
Nursing	1 (8.3%)
Healthcare Quality Improvement Coach	1 (8.3%)

## Data Availability

The data is unavailable due to ethical restrictions.
